# A Non-Linear Temperature Compensation Model for Improving the Measurement Accuracy of an Inductive Proximity Sensor and Its Application-Specific Integrated Circuit Implementation

**DOI:** 10.3390/s20175010

**Published:** 2020-09-03

**Authors:** Li Wang, Hui-Bin Tao, Hang Dong, Zhi-Biao Shao, Fei Wang

**Affiliations:** 1 The School of Electronic and Information Engineering, Xi’an Jiaotong University, No. 28, Xianning West Road, Xi’an 710049, China; wangliwnzz@stu.xjtu.edu.cn (L.W.); dhunter1230@gmail.com (H.D.); zbshao@mail.xjtu.edu.cn (Z.-B.S.); 2 The School of Software Engineering, Xi’an Jiaotong University, No.28, Xianning West Road, Xi’an 710049, China; coldfire2000@mail.xjtu.edu.cn

**Keywords:** inductive proximity sensor, wide temperature range, non-linear model, ASIC

## Abstract

The non-linear characteristic of a non-contacting Inductive Proximity Sensor (IPS) with the temperature affects the computation accuracy when measuring the target distance in real time. The linear model based method for distance estimation shows a large deviation at a low temperature. Accordingly, this paper presents a non-linear measurement model, which computes the target distance accurately in real time within a wide temperature range from −55 °C to 125 °C. By revisiting the temperature effect on the IPS system, this paper considers the non-linear characteristic of the IPS measurement system due to the change of temperature. The proposed model adopts a non-linear polynomial algorithm rather than the simple linear Look-Up Table (LUT) method, which provides more accurate distance estimation compared to the previous work. The introduced model is fabricated in a 0.18 μm Complementary Metal Oxide Semiconductor (CMOS) process and packaged in a CQFN40. For the most commonly used sensing distance of 4 mm, the computed distance deviation of the Application-Specific Integrated Circuit (ASIC) chips falls within the range of [−0.2,0.2] mm. According to the test results of the ASIC chips, this non-linear temperature compensation model successfully achieves real-time and high-accuracy computation within a wide temperature range with low hardware resource consumption.

## 1. Introduction

An Inductive Proximity Sensor (IPS) is a kind of tiny device deployed to measure the physical parameters of the surrounding area. The improvements of IPS began in the 1990s with the large-scale integration of the electronic components [1,2], playing an increasingly crucial role in civilian and other applications [3]. Most optical and capacitive sensors, despite demonstrating advantages in terms of resolution and accuracy, are generally sensitive to environmental changes [4,5]. In recent years, IPSs based non-contacting measurement systems have grown exponentially, especially in high-reliability applications, such as the medical field [6], the automotive industry [7], biomedical applications [8], and various industrial position sensing environments [9,10]. Many products are designed to meet temperature, vibration, and other requirements [11,12,13,14]. The requirement of high accuracy and easy ASIC implementation in industrial fields has led to continuous structural innovation for better linearity. A sigma-delta-type displacement-to-digital converter [15] is designed such that the digital output directly indicates the displacement that is being sensed, the results of which indicate a high degree of linearity. A novel noncontact inductive displacement sensor [16] is realized with a self-balancing signal conditioning circuit, whose output is linear to the displacement of the floating wiper. A novel timer based method for demodulating low-frequency amplitude-modulated (AM) signals [17] is proposed, which is discussed and evaluated in terms of ASIC implementation and the non-linearity. A non-contact inductive linear displacement sensor [18] is presented, which is based on planar coils realized by the Printed Circuit Board (PCB) technique. A non-contact displacement sensor employing a couple of planar coils etched on PCB and an E-type soft ferromagnetic core is proposed [19], which provides an output that varies linearly with the displacement. These innovations put more attention on improving the structure of the model. However, due to the inherent limitations of the material and structure, the functionality of the transducer suffers from temperature changes, which significantly impacts the measurement accuracy [20,21,22,23,24]. Permalloy based thin film structures [8] are proposed and studied in the temperature interval of 25 °C to 50 °C for biomedical applications. This paper focuses on the widely used sensors made of the copper coil rather than material optimization [8,25]. The authors of [26] proposed an automated measurement of the hysteresis of the temperature-compensated inductance-to-frequency converter with a single quartz crystal. Whereas the influence of the temperature drift is strongly reduced, the operational temperature range is limited from 0 °C to 50 °C. Furthermore, it increases the complexity of the structure, which adds two different inductances connected in series to the quartz crystal [26]. The measurement methods of [27,28] were proposed for online calculation with low hardware resources. Both of these methods achieve high accuracy at room temperature, but show large deviation at a low temperature. Since temperature is an important factor that decreases the estimation accuracy of the IPS measurement system, especially at a low temperature, a new method is required to compute the target distance quickly and accurately within a wide temperature range.

Focusing on the changing temperature, this paper takes a number of factors into consideration: (i) robustness to the temperature changes; (ii) high accuracy; (iii) easy ASIC implementation; (iv) flexible application. With these considerations, this paper presents a non-linear measurement model, which predicts the target distance quickly and accurately within a wide temperature range from −55 °C to 125 °C. Due to the significance of temperature, this paper revisits the temperature effect on the IPS measurement system and demonstrates the strong non-linear attributes when taking the temperature changes into consideration. Based on the analysis, the proposed model adopts a non-linear polynomial algorithm, which needs a parameter computation process to fit the non-linear curve. To ensure the high efficiency of the proposed IPS measurement model, we adopt a serial dual-stage scheme, in which the online stage estimates the target distance online with the parameters computed by the offline stage. Since the large amount of parameter computation can be performed in the offline stage, the efficiency of the online stage can be improved by reducing the amount of calculations and the complexity in ASIC chips. According to the obtained results of ASIC chips, this non-linear method manages to achieve real-time, high-accuracy computation within a wide temperature range.

The contributions of this work are summarized as follows:

1. The proposed model can perform an accurate estimation in real time within a wide temperature range.
2. We adopt a non-linear polynomial algorithm to improve the robustness for a wide temperature range, which provides more accurate distance estimation compared to the previous work.
3. We propose a serial dual-stage scheme to reduce the amount of online calculation and enable easy ASIC implementation.
4. The ASIC chips were taped out using 0.18 μm CMOS technology. Under the most commonly used sensing distance of 4 mm, the computed distance deviation of the ASIC chips falls within the range of [−0.2,0.2] mm. The success of the AISC implementation demonstrates the feasibility of the proposed non-linear model.

## 2. Revisiting the IPS Model

### 2.1. IPS Model

**Figure 1:**
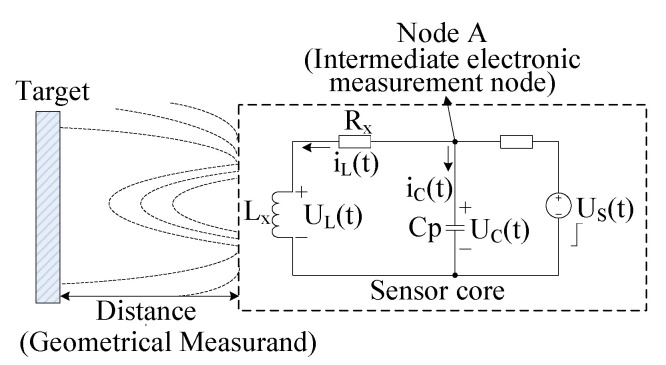
Block diagram of a classical IPS measurement circuit (the distance changes when the target travels between the target and the coil).

**Figure 2:**
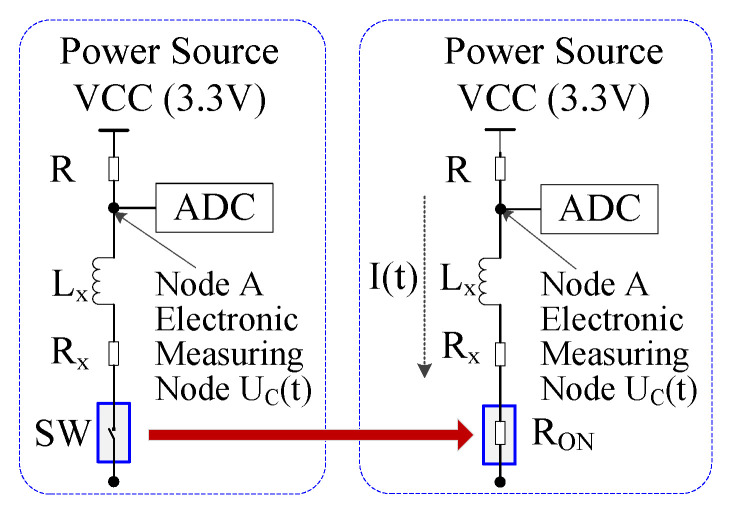
Theoretical IPS measurement circuit in simulation (the resistance $R_{\mathrm{ON}}$ caused by the actual switching (SW) operation is considered).

In order to analyze the classical IPS measurement circuit (Figure 1) accurately, the resistance caused by the actual switching operation is considered (SW in Figure 2) for the theoretical IPS measurement circuit in our simulation.

In Figure 1, the target travels between the target and the coil, resulting in the change of the distance and the inductance $L_{x}$. The value of $L_{x}$ affects the electronic measurement at Node A, which demonstrates the non-linear relationship to the physical target distance. By driving with a voltage source of $U_{s}\left(t\right)$ and measuring at Node A, the target distance can be provided. $C_{p}$ is the equivalent distributed capacitance; $L_{x}$ is the equivalent distributed inductance; R is the current limitation resistance; $R_{x}$ is the resistance of the inductor.

However, when the resistances ($R_{x}$ and R) are affected by the changing temperature, the electronic output signal at Node A changes accordingly, which increases the deviation of the target distance estimation. Thus, due to the changing temperature, revisiting the IPS model is necessary.

In Figure 2, a 12 bit Analog-to-Digital Converter (ADC) is used with a power source VCC (VCC=3.3 V). R is the current limitation resistance; $R_{x}$ is the resistance of the inductor; and $R_{\mathrm{ON}}$ is the resistance of the switch. $K_{R}$, $K_{\mathrm{Rx}}$, and $K_{\mathrm{RON}}$ are the Temperature Coefficients (TCs) of resistances R, $R_{x}$, and $R_{\mathrm{ON}}$ respectively, shown in Table 1. In this paper, the room temperature $t_{0}=25\;{}^{\circ}C$. t is the current temperature, which changes from −55 °C to 125 °C in use. $R_{0}$, $R_{x0}$, and $R_{\mathrm{ON}0}$ are the typical values of R, $R_{x}$, and $R_{\mathrm{ON}}$ at room temperature, respectively, shown in Table 2. Based on the working voltage (VCC=3.3 V), the limitation of dynamic power consumption (I(t)<15 mA), and the current limitation of the low-power high-speed MOS switch, the working current of the coil is determined to be 12 mA, after which the current limiting resistance $R_{0}$ can be deduced as 250 Ω. The coil is made of AWG41 wire (d = 0.071 mm), with a wire length of about 6 m, leading to an $R_{x0}$ resistance of 22 Ω at room temperature. Based on resistance of the 2N7002 transistor at room temperature and the working electrical parameters (the voltage between gate and source $V_{\mathrm{gs}}$, the drain current $I_{d}$), after the actual test of the on-state voltage $V_{\mathrm{dson}}$ ($I_{d}$ = 12 mA, $V_{\mathrm{dson}}$ = 0.444 V), $R_{\mathrm{ON}0}$ is calculated as 3.7 Ω.

### 2.2. Temperature Effect

#### 2.2.1. Linear Change of Resistance

In the theoretical IPS measurement circuit of Figure 2, the resistance R, $R_{x}$, and $R_{\mathrm{ON}}$ change linearly with the temperature t, shown in Equation (1). Due to this linear change of resistance, the output voltage $U_{C}\left(t\right)$ at node A also changes with the temperature.

(1) $$\{\begin{array}{c} R\left(t\right)=R_{0}\times \left[1+K_{R}\times \left(t-t_{0}\right)\right]\\ R_{x}\left(t\right)=R_{x0}\times \left[1+K_{\mathrm{Rx}}\times \left(t-t_{0}\right)\right]\\ R_{\mathrm{ON}}\left(t\right)=R_{\mathrm{ON}0}\times \left[1+K_{\mathrm{RON}}\times \left(t-t_{0}\right)\right] \end{array}.$$

#### 2.2.2. Non-Linear Change of Voltage

The linear change of resistance with the temperature brings the non-linear change of the voltage $U_{C}$. Thus, the changing temperature increases the computation deviation of distance estimation in linear LUT computation.

Besides the changing temperature, the computation amount is another necessary consideration in this paper. Equation (2) shows the solution of the classical system in Figure 1 [28]. It can easily be seen that $U_{C}$ has a complex non-linear relationship with temperature t, which is too complex to be solved directly in ASIC. Because temperature is an important and necessary contributor in the IPS system, it is necessary to propose a simplified method for accurate online computation.

(2) $$\{\begin{array}{c} U_{C}\left(t_{\mathrm{time}}\right)=A_{1}\left(t\right)e^{p_{1}\left(t\right)t_{\mathrm{time}}}+A_{2}\left(t\right)e^{p_{2}\left(t\right)t_{\mathrm{time}}}+\frac{R_{x(t)}}{R_{x}\left(t\right)+R\left(t\right)}U_{S}\left(0^{+}\right)\\ A_{1}\left(t\right)=\frac{\frac{1}{R\left(t\right)C_{p}}+p_{2}\frac{R_{x}\left(t\right)}{R\left(t\right)+R_{x}\left(t\right)}}{p_{1}-p_{2}}U_{S}\left(0^{+}\right)\\ A_{2}\left(t\right)=\frac{\frac{1}{R\left(t\right)C_{p}}+p_{1}\frac{R_{x}\left(t\right)}{R\left(t\right)+R_{x}\left(t\right)}}{p_{2}-p_{1}}U_{S}\left(0^{+}\right)\\ p_{1}\left(t\right)=\frac{-R\left(t\right)C_{p}R_{x}\left(t\right)-L_{x}+\sqrt{{\left(R\left(t\right)C_{p}r_{a}\left(t\right)+L_{x}\right)}^{2}-4R\left(t\right)C_{p}L_{x}\left(R\left(t\right)+R_{x}\left(t\right)\right)}}{2R\left(t\right)C_{p}L_{x}}\\ p_{2}\left(t\right)=\frac{-R\left(t\right)C_{p}R_{x}\left(t\right)-L_{x}+\sqrt{{\left(R\left(t\right)C_{p}r_{a}\left(t\right)+L_{x}\right)}^{2}-4R\left(t\right)C_{p}L_{x}\left(R\left(t\right)+R_{x}\left(t\right)\right)}}{2R\left(t\right)C_{p}L_{x}} \end{array}.$$

Sampling $U_{C}$ in Equation (2) at time $t_{\mathrm{time}}=t_{2}$ and $t_{\mathrm{time}}=t_{1}$ [28], we can obtain $U_{2}$, $U_{1}$ as follows:

(3) $$\{\begin{array}{c} U_{2}\left(t\right)=\frac{R_{x}\left(t\right)}{R_{x}\left(t\right)+R\left(t\right)}\\ U_{1}\left(t\right)=A_{1}\left(t\right)e^{p_{1}\left(t\right)t_{1}}+A_{2}\left(t\right)e^{p_{2}\left(t\right)t_{1}}+\frac{R_{x}\left(t\right)}{R_{x}\left(t\right)+R\left(t\right)}U_{S}\left(0^{+}\right) \end{array}.$$

$U_{1}$ shows the same characteristic as $U_{C}$ regarding the temperature and computation amount. To analyze the temperature effect and computational complexity, we use two methods: the theoretical method for the model in Figure 2 and the linear LUT based method in [28].

**Figure 3:**
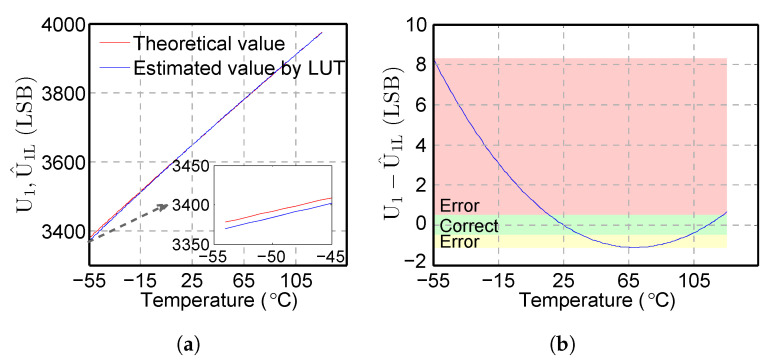
(**a**) Theoretical U_1_ and computed value of ${\hat{U}}_{1L}$ by the LUT method; (**b**) estimation deviation of the LUT method $\Delta U_{1L}=U_{1}-{\hat{U}}_{1L}$. The deviation $\Delta U_{1L}\geq 0.5\;\mathrm{LSB}$ (red area) and $\Delta U_{1L}\leq -0.5\;\mathrm{LSB}$ (yellow area) result in an incorrect voltage estimation. The LUT method shows a large estimation deviation at a low temperature.

The comparison between the computed $U_{1}$ by the theoretical method and the estimated ${\hat{U}}_{1L}$ by the LUT method in [28] when $L_{x}=5\;\mathrm{mH}$ is shown in Figure 3. The average value of $\Delta U_{1L}=U_{1}-{\hat{U}}_{1L}$ is 1.2 LSB, which causes the deviation of the voltage estimation.

In the red area of Figure 3b, when $-55{\;}^{{}^{\circ}}C\leq t\leq 16{\;}^{{}^{\circ}}C$, $\Delta U_{1L}\geq 0.5\;\mathrm{LSB}$. This means that ${\hat{U}}_{1L}$ is 0.5 LSB larger than $U_{1L}$, which results in an incorrect voltage $U_{1L}$ estimation. In the green area, when $17{\;}^{{}^{\circ}}C\leq t\leq 36{\;}^{{}^{\circ}}C$ and $102{\;}^{{}^{\circ}}C\leq t\leq 125{\;}^{{}^{\circ}}C$, $\Delta U_{1L}\leq 0.5\;\mathrm{LSB}$. In this case, the estimated ${\hat{U}}_{1L}$ is very close to $U_{1L}$, which means the method [28] works correctly. In the yellow area, $37{\;}^{{}^{\circ}}C\leq t\leq 101{\;}^{{}^{\circ}}C$, $\Delta U_{1L}\leq -0.5\;\mathrm{LSB}$. ${\hat{U}}_{1L}$ is −0.5 LSB less than $U_{1}$, which also results in an incorrect voltage estimation. It can further be seen clearly in Figure 3 that the estimation deviation of ${\hat{U}}_{1L}$ is large at a low temperature, i.e., almost 8 LSB at $-55{\;}^{{}^{\circ}}C$.

The analysis shown in Figure 3 provides the temperature range for the proposed method in Section 3.2.2. Based on this analysis of the temperature range, the proposed method could purposefully increase the computation accuracy and reduce the amount of calculation.

#### 2.2.3. Inductance Change

In addition to the above IPS internal parameters, the inductance $L_{x}$, which is decided by the physical target distance, also influences the value of the measured voltage $U_{C}\left(t\right)$. The solution of the theoretical system in Figure 2 is as follows:

(4) $$\{\begin{array}{c} U_{C}\left(t\right)=V_{\mathrm{cc}}-R\left(t\right)\times I\left(t\right)\\ R_{\mathrm{ALL}}\left(t\right)=R\left(t\right)+R_{x}\left(t\right)+R_{\mathrm{ON}}\left(t\right)\\ I\left(t\right)=\frac{V_{\mathrm{cc}}}{R_{\mathrm{ALL}}\left(t\right)}\left(1-e^{-\frac{t_{\mathrm{time}}}{\tau (t)}}\right)\\ \tau \left(t\right)=\frac{L_{x}}{R_{\mathrm{ALL}}\left(t\right)} \end{array}.$$

where $U_{C}\left(t\right)$ is the measured voltage, whose value is related to the present estimated distance at current temperature t. When the inductance $L_{x}$ changes from 4.5 mH to 5.5 mH, the simulation results show that the computation deviation between the theoretical value $U_{1L}$ and the estimated ${\hat{U}}_{1L}$ by the LUT method is affected by the temperature, but not the distance-related parameter $L_{x}$.

#### 2.2.4. ADC and LDO Change

**Figure 4:**
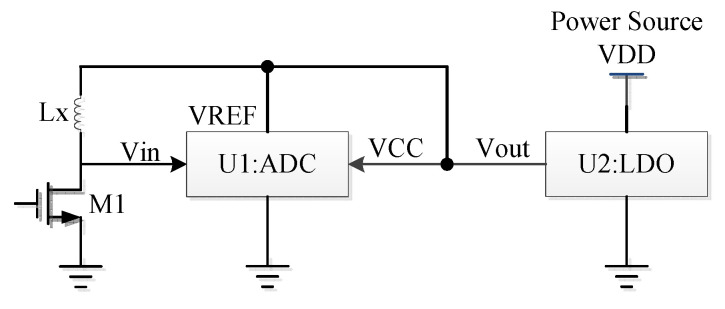
Block diagram of the ADC and LDO circuit (we propose methods to reduce the influence of the temperature).

When measuring the voltage $U_{1}$, we propose the following methods in the ADC circuit to reduce the influence of the temperature, shown in Figure 4.

1. The output voltage $V_{\mathrm{out}}$ of the LDO supplies the voltage as the input voltage Vin of the ADC for the charge-discharge circuit of the IPS, the reference voltage VREF of the ADC, and the supply voltage VCC of the ADC. This kind of connection for the voltage supplies reduces the temperature effect on measuring $U_{1}$.
2. The ADC is redesigned with the optimized temperature coefficients for gain and offset.

The test results show that the deviations of $U_{2}$ and $U_{1}$ are about 2–3 LSB due to the circuit of the ADC and LDO when the temperature changes. The deviation caused by the circuit of the ADC and LDO is smaller than that by the resistances in Section 2.2.1. Moreover, the innovation of the ADC is another improvement activity in the analog field.

### 2.3. Summary of This Section

The conclusions drawn from the above analysis and simulation can be summarized as follows:

1. The complexity of the $U_{C}$ equation requires a method with high accuracy, but not too much online computation work.
2. The resistances R, $R_{x}$, and $R_{\mathrm{ON}}$ change linearly with the temperature t, which results in the non-linear change of the voltages $\left\{U_{2},U_{1}\right\}$ and the computation deviation of distance estimation.
3. The inductance $L_{x}$ from 4.5 mH to 5.5 mH (or the target distance) has little effect on the computation deviation of ${\hat{U}}_{1}$.
4. The temperature plays a significant role in computing $\left\{U_{2},U_{1}\right\}$ and estimating the physical distance. The LUT based method in [28] largely simplifies the calculation, but produces a large estimation deviation at a low temperature.

## 3. Proposed Method

Due to the limitation of hardware resources, it is almost impossible to compute the $U_{C}$ and estimate the target distance directly using Equation (4). Although the linear measurement methods such as the LUT method in [28] could largely simplify the calculation, they produce a large estimation deviation due to the temperature effect, especially at a low temperature.

The overall goal of this study is to successfully realize a real-time and high-accuracy measurement method within a wide temperature range. The proposed method contains an offline and an online stage to achieve computation reduction, easy portability, robustness to the temperature changes, and easy ASIC implementation.

### 3.1. The Dual-Stage Scheme

**Figure 5:**
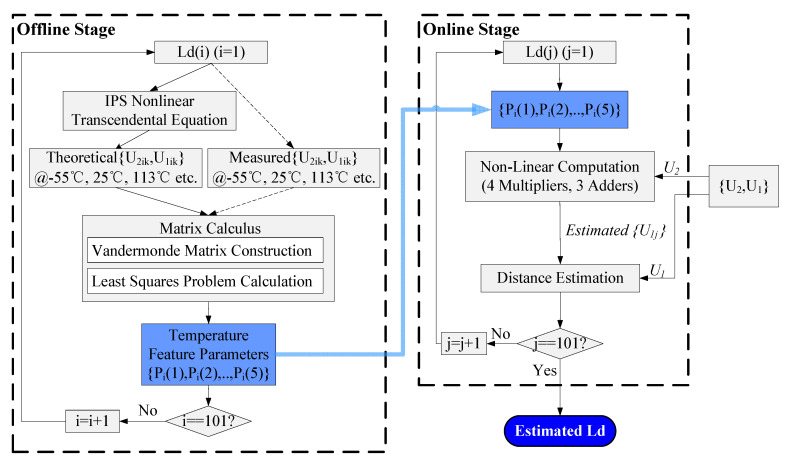
Block diagram of the dual-stage scheme (the offline stage performs a large amount of computation and generates the temperature feature parameters, while the online stage predicts the target distance based on a non-linear piecewise fitting scheme with these parameters).

The dual-stage scheme includes an offline stage and an online stage, as shown in Figure 5. The offline stage performs a large amount of computation based on the theoretical or measured voltages of $\left\{U_{2\mathrm{ik}},U_{1\mathrm{ik}}\right\}$ and generates the temperature feature parameters $\left\{P_{i}\left(1\right),P_{i}\left(2\right),\ldots ,P_{i}\left(5\right)\right\}$, which are used in the online stage. Since these calculations do not exist in the ASIC, the amount of online calculation in the ASIC is significantly reduced with little hardware resource.

The online stage predicts the target distance based on a non-linear piecewise fitting scheme with the temperature feature parameters from the offline stage. Based on the voltages $\left\{U_{2\mathrm{ik}},U_{1\mathrm{ik}}\right\}$, (i=1,…,101;k=1,…,6) introduced in Equation (3), the offline stage generates 5 temperature feature parameters for each distance $L_{d}\left(i\right),\;\left(i=1,\ldots ,101\right)$. These parameters are stored in the system and used in the online stage.

In Figure 5, the subscript {i,j}, (i,j=1,…,101) represents each distance point from 3 mm to 7 mm with a step of 0.04 mm.

### 3.2. The Offline Stage

#### 3.2.1. The Temperature Feature Parameters

The temperature feature parameters represent the complex relationship between $U_{2}$ and $U_{1}$, which can be calculated in an offline manner to reduce the amount of calculation and complexity of the ASIC. For each distance $L_{d}$, the offline stage uses $\left\{U_{2\mathrm{ik}},U_{1\mathrm{ik}}\right\}$ voltage pairs to calculate five feature parameters at six temperature points: $-55{\;}^{{}^{\circ}}C,-31{\;}^{{}^{\circ}}C,\;-7{\;}^{{}^{\circ}}C,\;16{\;}^{{}^{\circ}}C,\;25{\;}^{{}^{\circ}}C$, and $101{\;}^{{}^{\circ}}C$.

To evaluate the algorithm comprehensively and objectively, two kinds of voltages are used in this paper: theoretical $\left\{U_{2\mathrm{ik}},U_{1\mathrm{ik}}\right\}$ in the lab simulation step and the measured values in the practical application. All temperature feature parameters $\{P_{i}\left(1\right),P_{i}\left(2\right),\ldots ,P_{i}\left(5\right)\},\left(1,\ldots ,101\right)$ are stored as constants in the ASIC. Thus, the massive offline matrix calculus in the offline stage only uses the storage space in the ASIC, but it does not bring computational burden to the online stage.

Simulation results are introduced in Section 5.1, in which theoretical $\left\{U_{2\mathrm{ij}},U_{1\mathrm{ij}}\right\}$ are generated by means of non-linear Equation (4). The ASIC results are presented in Section 5.2, in which the measured $\left\{U_{2\mathrm{ij}},U_{1\mathrm{ij}}\right\}$ are actually measured voltage values.

#### 3.2.2. Piecewise Fitting

According to Figure 3b, the estimated voltage ${\hat{U}}_{1L}$ of the linear method shows distinct characteristics regarding the temperature t. The following can be obtained:

1. When $-55{\;}^{{}^{\circ}}C\leq t\leq 16{\;}^{{}^{\circ}}C$, the computed ${\hat{U}}_{1L}$ in the linear method exhibits a larger deviation from the theoretical value $U_{1}$, indicating the obvious non-linear characteristic of the IPS system.
2. When $17{\;}^{{}^{\circ}}C\leq t\leq 125{\;}^{{}^{\circ}}C$, the small deviation between ${\hat{U}}_{1L}$ and $U_{1}$ indicates the linear characteristic of the IPS system.
3. $U_{1}-{\hat{U}}_{1L}\approx 0$ at temperature points $t=25{\;}^{{}^{\circ}}C$ and $t=101{\;}^{{}^{\circ}}C$ indicates accurate voltage estimation.

According to the above conclusions, we propose a temperature based piecewise fitting scheme to compute the temperature feature parameters at the different temperature ranges according to their corresponding voltage deviation. With this fitting scheme, the proposed model can better represent the IPS measurement system when the temperature is changing, which could improve the estimation accuracy.

In the low temperature range of $-55{\;}^{{}^{\circ}}C\leq t\leq 16{\;}^{{}^{\circ}}C$, the matrix calculus module computes the coefficients of a polynomial $P_{i}\left(\left\{U_{2i1},U_{2i2},U_{2i3},U_{2i4}\right\}\right)$ with a higher degree N (N=2) that is a best fit (in a least squares sense) for the data of $\left\{U_{1i1},U_{1i2},U_{1i3},U_{1i4}\right\}$. $\left\{U_{1i1},U_{2i1}\right\}$, $\left\{U_{1i2},U_{2i2}\right\}$, $\left\{U_{1i3},U_{2i3}\right\}$, and $\left\{U_{1i4},U_{2i4}\right\}$ are the voltage values at a temperature of $-55{\;}^{{}^{\circ}}C$, $-31{\;}^{{}^{\circ}}C$, $-7{\;}^{{}^{\circ}}C$, and $16{\;}^{{}^{\circ}}C$ respectively; that is:

(5) $$\left\{U_{1i1},U_{1i2},U_{1i3},U_{1i4}\right\}=P_{i}\left(\left\{U_{2i1},U_{2i2},U_{2i3},U_{2i4}\right\}\right),i=1,\ldots ,101.$$

Moreover, in the temperature range of $17{\;}^{{}^{\circ}}C\leq t\leq 125{\;}^{{}^{\circ}}C$, the matrix calculus module computes with a lesser degree N (N=1) based on voltage pairs at $25{\;}^{{}^{\circ}}C$ and $113{\;}^{{}^{\circ}}C$; that is:

(6) $$\left\{U_{1i1},U_{1i2}\right\}=P_{i}\left(\left\{U_{2i1},U_{2i2}\right\}\right),i=1,\ldots ,101.$$

**Figure 6:**
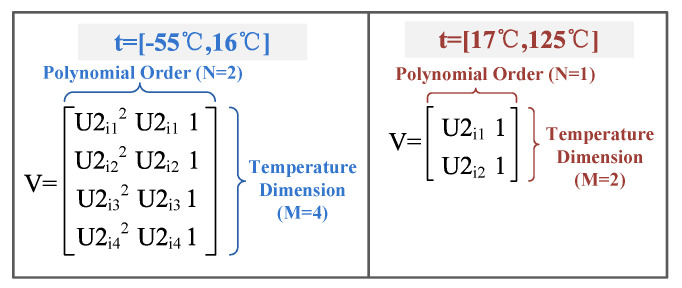
Structure of the Vandermonde matrix (in the low temperature range of $-55{\;}^{{}^{\circ}}C\leq t\leq 16{\;}^{{}^{\circ}}C$, the matrix module computes with a higher degree N=2, while in the temperature range of $17{\;}^{{}^{\circ}}C\leq t\leq 125{\;}^{{}^{\circ}}C$, the matrix module computes with a lesser degree N=1).

The matrix calculus module includes two parts: Vandermonde matrix construction and least squares problem calculation. The dimension of the Vandermonde matrix is [M×N], where M represents the number of temperature points included and N is the polynomial order plus one, as shown in Figure 6.

The least squares problem calculation conducts the orthogonal-triangular decomposition, and it decomposes matrix V into a product of an M-by-N upper triangular matrix R and an M-by-N unitary matrix Q, so that V=Q×R. When N=2, the polynomial coefficients $\left\{P_{i}\left(1\right),P_{i}\left(2\right),P_{i}\left(3\right)\right\}$ in descending powers are computed by $R\backslash Q^{\prime }\times \left\{U1_{i1},U1_{i2},U1_{i3},U1_{i4}\right\}$. When N=1, the polynomial coefficients $\left\{P_{i}\left(4\right),P_{i}\left(5\right)\right\}$ in descending powers are computed by $R\backslash Q^{\prime }\times \left\{U1_{i1},U1_{i2}\right\}$.

Because the temperature feature parameters are calculated offline, the complexity of the computation does not increase the hardware resource consumption for the ASIC implementation. After moving all locations of the specified distance points $L_{d}\left(i\right),\left(i=1,\ldots ,101\right)$, the offline stage generates the temperature feature parameters, as shown in Table 3.

### 3.3. The Online Stage

The introduced online stage aims to predict the target distance with high accuracy and minimal hardware consumption. By means of the non-linear piecewise computation, the online stage first generates the voltage pool of $\left\{U_{1j}\right\},\left(j=1,\ldots ,101\right)$ using the temperature feature parameters from the offline stage. Then, the online stage performs the online distance estimation, which predicts the target distance in real time with little storage resource.

#### 3.3.1. Voltage Pool

Based on the temperature feature parameters, the online stage generates the voltage pool by means of the non-linear piecewise computation. The piecewise computation scheme performs both linear and non-linear polynomial calculations according to the temperature range, which could improve computation accuracy and reduce the unnecessary calculations simultaneously.

To better illustrate the piecewise computation scheme, we use the input of voltage group $\left\{U_{2},U_{1}\right\}$ as the testing point, which is either computed by theoretical Equation (4) in simulation or measured in field tests. For each j, based on the theoretical or measured voltage $U_{2}$, the non-linear computation module computes the voltage pool of $\left\{{\hat{U}}_{1j}\right\}$ online by the pre-calculated $\{P_{j}\left(1\right),P_{j}\left(2\right),\ldots ,P_{j}\left(5\right)\}$, which uses the time-sharing hardware resources of four multipliers and three adders. These hardware resources are repeatedly used 101 times to cover the distance range of [3 mm,7 mm]. At a low temperature range from $-55{\;}^{{}^{\circ}}C$ to $16{\;}^{{}^{\circ}}C$, the piecewise computation method computes the voltage pool with a higher degree N (N=2) based on $\{P_{j}\left(1\right),P_{j}\left(2\right),P_{j}\left(3\right)\}$. For the high temperature range of from $17{\;}^{{}^{\circ}}C$ to $125{\;}^{{}^{\circ}}C$, the method computes with a lesser degree N (N=1) based on $\{P_{j}\left(4\right),P_{j}\left(5\right)\}$.

As illustrated in Equation (3), the voltage $U_{2}$ is only related to the temperature. According to the simulation results, we assume $U_{2j}\leq 900$ can represent the temperature range $t=[-55{\;}^{{}^{\circ}}C,16{\;}^{{}^{\circ}}C]$. Therefore, the online computation of the voltage pool $\left\{{\hat{U}}_{1j}\right\},\left(j=1,\ldots ,101\right)$ for each time-sharing clip is as follows:

(7) $${\hat{U}}_{1}\left(j\right)=\{\begin{array}{c} P_{j}\left(1\right)\times U_{2j}^{2}+P_{j}\left(2\right)\times U_{2j}+P_{j}\left(3\right),U_{2j}\leq 900\\ P_{j}\left(4\right)\times U_{2j}+P_{j}\left(5\right) \end{array}.$$

#### 3.3.2. Distance Estimation

The distance estimation module chooses the voltage value from the voltage pool of $\left\{{\hat{U}}_{1j}\right\}$ that is closest to the current measured $U_{1}$, and the sequence number of this chosen voltage represents the estimated distance ${\hat{L}}_{d}$.

Specifically, the distance estimation module computes the deviation of $\Delta {\hat{U}}_{1}\left(j\right)=|{\hat{U}}_{1}\left(j\right)-U_{1}|$ and compares $\Delta {\hat{U}}_{1}\left(j\right)$ with the previously stored $\Delta U_{1\min}$. If $\Delta {\hat{U}}_{1}\left(j\right)<\Delta U_{1\min}$, $\left\{\Delta U_{1\min},\;j_{\mathrm{OUT}}\right\}$ is replaced by $\left\{\Delta {\hat{U}}_{1}\left(j\right),\;j\right\}$; otherwise, $\left\{\Delta U_{1\min},\;j_{\mathrm{OUT}}\right\}$ is kept unchanged. The initial value of $\left\{\Delta U_{1\min},\;j_{\mathrm{OUT}}\right\}$ is defined as $\left\{\Delta U_{1}\left(1\right),\;1\right\}$. The distance mark $j_{\mathrm{OUT}}$ is the sequence number of $\Delta U_{1\min}$. After 101 cycles, the scheme outputs the distance mark $j_{\mathrm{OUT}}$ and its corresponding distance value $[3+\left(j_{\mathrm{OUT}}-1\right)\times 0.004]\;\mathrm{mm}$. In other words, the distance estimation module searches from $\left\{{\hat{U}}_{1}\left(j\right),\;k=1,\ldots ,101\right\}$, which are computed online based on the input of $U_{2}$, and then finds the value that is nearest to the input of $U_{1}$.

### 3.4. Summary of This Section

To achieve high accuracy and easy ASIC implementation, the proposed method adopts the following:

1. The dual-stage scheme: This scheme includes an offline stage and an online stage, which enables computation reduction, flexible application, strong temperature adaptability, and easy ASIC realization.
2. The offline calculation of the temperature feature parameters: These parameters can be calculated in an offline manner to reduce the amount of calculation and complexity significantly when computing $U_{2}$ and $U_{1}$ under the changing temperature.
3. The piecewise fitting scheme: This scheme performs the piecewise fitting at the different temperature ranges according to their corresponding voltage deviation, which makes the proposed model close to the IPS measurement system when the temperature is changing.
4. The voltage pool generated by the online piecewise computation: The online stage generates the voltage pool by means of the non-linear piecewise computation. The temperature based piecewise computation performs both linear and non-linear polynomial online calculation according to the temperature range, which could improve the computation accuracy and reduce the unnecessary calculation simultaneously.
5. The distance estimation: By choosing the optimal voltage value, the proposed model can predict the target distance in real time.

Considering the non-linear characteristic of the IPS measurement system with the change of temperature, the proposed method could successfully achieve real-time and high-accuracy target estimation under the changing temperature with low hardware resource consumption. The simulation and ASIC results are shown in Section 5.

## 4. ASIC Implementation

Based on the stage technology of the proposed algorithm, this product was already released in 2019 for industrial fields such as landing gears.

**Figure 7:**
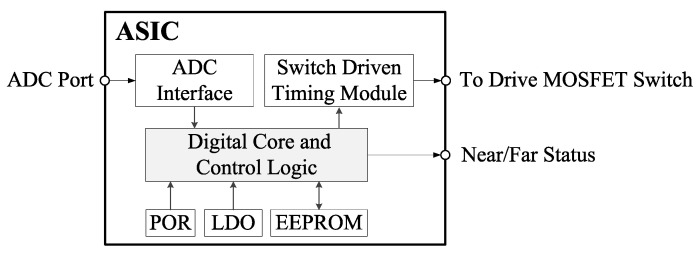
Functional block diagram of the ASIC (AD mixed CMOS integrated circuit).

This product is implemented in an Analog Digital (AD) mixed CMOS integrated circuit, which includes the digital stage of the proposed algorithm, the Electrically Erasable Programmable Read Only Memory (EEPROM), the Low Dropout Regulator (LDO), the ADC interface logic, and the Power On Reset (POR) circuit, as shown in Figure 7.

**Figure 8:**
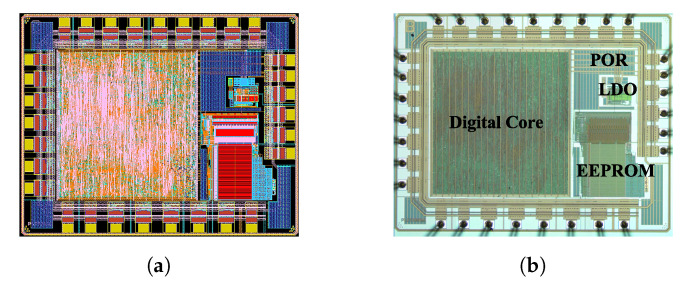
(**a**) Layout; (**b**) die micrograph. The die size is 3.2 mm × 2.7 mm.

The die layout in Figure 8a mirrors the schematic representation of the architecture. Figure 8b is a die micrograph. The die size is 3.2 mm×2.7 mm.

**Figure 9:**
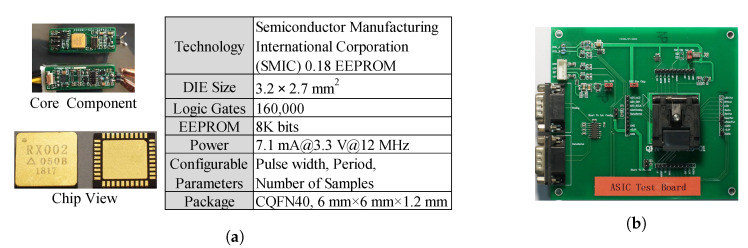
(**a**) Chip info (the ASIC chip is fabricated in the 0.18 μm CMOS process and encapsulated in a 40-pin CQFN); (**b**) evaluation PCB board (the size of the board is 10 mm × 25 mm).

The ASIC chip is fabricated in the 0.18 μm CMOS process and encapsulated in a 40-pin CQFN, 6 mm×6 mm×1.2 mm (Figure 9a). This chip is mounted on a PCB circuit as a core component (Figure 9a), which is then integrated into the 10 mm×25 mm evaluation PCB board (Figure 9b).

## 5. Results and Discussion

Both the theoretical simulation and ASIC results are conducted to evaluate the accuracy of the proposed model in this section. It is noted that the inductance $L_{x}$, instead of the distance $L_{d}$, is used in the theoretical mode (Figure 2) and is inversely proportional to $L_{d}$, thus making $L_{x}$ an appropriate metric in the theoretical simulation.

### 5.1. Simulation Results

As shown in (Figure 2), the value of $L_{x}$ changes when the target travels, resulting in the change of $U_{1}$. Therefore, we evaluate $U_{1}$ and $L_{x}$ in the theoretical simulation of this subsection.

#### 5.1.1. Simulation of $U_{1}$

First, the intermediate variable $U_{1}$ is analyzed in the simulation. We use the difference of the voltage ${\hat{U}}_{1}$ to evaluate the performance of the proposed model. Specifically, we define $\Delta {\hat{U}}_{1L}$ as the difference between $U_{1}$ of the theoretical Equation (4) and ${\hat{U}}_{1L}$ of the LUT method [28] and $\Delta {\hat{U}}_{1P}$ as the difference between $U_{1}$ of the theoretical Equation (4) and ${\hat{U}}_{1P}$ of the method proposed in this paper, such that:

(8) $$\{\begin{array}{c} \Delta {\hat{U}}_{1L}\left(i,j\right)=U_{1}\left(i,j\right)-{\hat{U}}_{1L}\left(i,j\right)\\ \Delta {\hat{U}}_{1P}\left(i,j\right)=U_{1}\left(i,j\right)-{\hat{U}}_{1P}\left(i,j\right) \end{array}.$$

**Figure 10:**
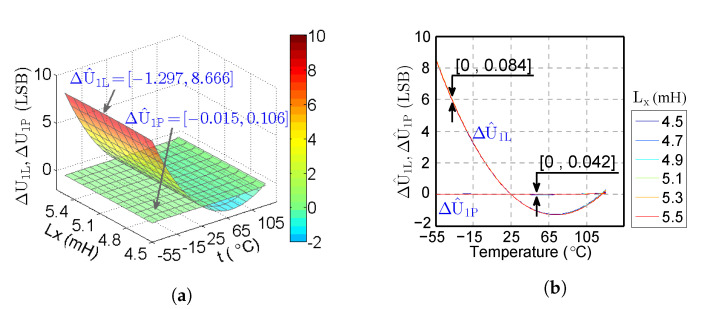
$\Delta {\hat{U}}_{1L}$ and $\Delta {\hat{U}}_{1P}$ with different $L_{x}$ displayed by: (**a**) 3D map (the estimated deviation $\Delta {\hat{U}}_{1P}$ of our method is much less than the deviation $\Delta {\hat{U}}_{1L}$ by LUT, especially at a low temperature); (**b**) 2D map (at each temperature point, the maximum of $\Delta {\hat{U}}_{1P}$ is less than $\Delta {\hat{U}}_{1L}$ with different $L_{x}$).

When the target moves in the IPS system, the inductance $L_{x}$ changes accordingly. Here, the subscript i represents the sequence of inductance from 4.5 mH to 5.5 mH with a step of 0.01 mH and the subscript j represents the temperature range from $-55{\;}^{{}^{\circ}}C$ to $125{\;}^{{}^{\circ}}C$ with a step of $1{\;}^{{}^{\circ}}C$. To analyze the calculation accuracy of $U_{1}$, $\Delta {\hat{U}}_{1L}$ and $\Delta {\hat{U}}_{1P}$ are shown in Figure 10. The shape of $\Delta {\hat{U}}_{1L}$ is curved from −1.297 LSB to 8.666 LSB. The shape of $\Delta {\hat{U}}_{1P}$ is almost a plane of zero value, whose range is [−0.015,0.106] LSB. $\Delta {\hat{U}}_{1P}$ is much less than $\Delta {\hat{U}}_{1L}$, which demonstrates that the proposed model reduces the estimated deviation of $U_{1}$. At each temperature point, the maximum of $\Delta {\hat{U}}_{1L}$ with different $L_{x}$ is 0.084 LSB, whereas this value is 0.042 LSB for $\Delta {\hat{U}}_{1P}$. Thus, the estimated deviation of $U_{1}$ caused by the changing inductance is minimal and can be ignored. Table 4 lists the detailed data analysis of $\Delta {\hat{U}}_{1L}$ and $\Delta {\hat{U}}_{1P}$. $\Delta {\hat{U}}_{1P}$ is reduced by about two orders of magnitude of $\Delta {\hat{U}}_{1L}$.

Therefore, we can conclude that all the computed ${\hat{U}}_{1P}$ are closer to the theoretical $U_{1}$ than ${\hat{U}}_{1L}$ for all $L_{x}$ in the simulation, and thus, the proposed model improves the calculation accuracy of $U_{1}$ over the LUT method in the theoretical simulation.

#### 5.1.2. Simulation of $L_{x}$

Based on the calculation of the voltage $U_{1}$, the output inductance of $L_{x}$ can be estimated. When the inductance changes from 4.5 mH to 5.5 mH with a step of 1 μH and the temperature changes from $-55{\;}^{{}^{\circ}}C$ to $125{\;}^{{}^{\circ}}C$ with a step of $1{\;}^{{}^{\circ}}C$ in the simulation, the number of test cases is 1001×181= 181,181. Equation (9) illustrates the computed inductance $\Delta {\hat{L}}_{\mathrm{xL}}$ and $\Delta {\hat{L}}_{\mathrm{xP}}$, that is:

(9) $$\{\begin{array}{c} \Delta {\hat{L}}_{\mathrm{xL}}\left(i,j\right)=L_{x}\left(i,j\right)-{\hat{L}}_{\mathrm{xL}}\left(i,j\right)\\ \Delta {\hat{L}}_{\mathrm{xP}}\left(i,j\right)=L_{x}\left(i,j\right)-{\hat{L}}_{\mathrm{xP}}\left(i,j\right) \end{array}.$$

where the subscript i=1,…,1001 represents the sequence of inductance, the subscript j=1,…,181 represents the sequence of temperature, $L_{x}$ is the theoretical inductance value, ${\hat{L}}_{\mathrm{xL}}$ is the estimated inductance by the LUT method, and ${\hat{L}}_{\mathrm{xP}}$ is the estimated value by the proposed method.

**Figure 11:**
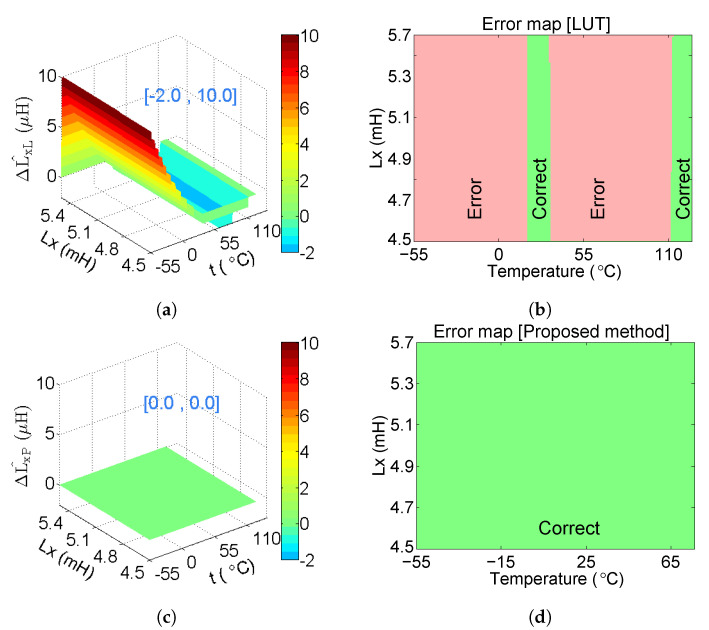
(**a**) ${\hat{L}}_{\mathrm{xL}}$ with different $\left\{L_{x},\;t\right\}$ (${\hat{L}}_{\mathrm{xL}}$ exhibits a large deviation under the low temperature range); (**b**) error map of ${\hat{L}}_{\mathrm{xL}}$ (the green areas indicate the correct computation that ${\hat{L}}_{\mathrm{xL}}=L_{x}$ and the red areas indicate the incorrect computation that ${\hat{L}}_{\mathrm{xL}}\neq L_{x}$); (**c**) ${\hat{L}}_{\mathrm{xL}}$ with different $\left\{L_{x},\;t\right\}$ (${\hat{L}}_{\mathrm{xL}}=0$ demonstrates that the deviation is reduced obviously); (**d**) error map of ${\hat{L}}_{\mathrm{xL}}$ (all the areas are green, indicating that the proposed method can obtain correct results for all the estimations).

In Figure 11, $\Delta {\hat{L}}_{\mathrm{xL}}\subseteq [-2,10]$ μH and $\Delta {\hat{L}}_{\mathrm{xP}}\subseteq \left[0\right]$ μH, which demonstrates that the deviation of the computed inductance is reduced obviously. As can be seen in Figure 11, ${\hat{L}}_{\mathrm{xL}}$ exhibits a large deviation under the low temperature range. In Figure 11b, the green areas indicate the correct computation that ${\hat{L}}_{\mathrm{xL}}=L_{x}$, and the red areas indicate the incorrect computation that ${\hat{L}}_{\mathrm{xL}}\neq L_{x}$. Since all the areas in Figure 11d are green, the proposed method can obtain correct results for all the estimations. Compared to the LUT method, the proposed model can reduce the error rate from 84.3% to 0%, which demonstrates the effectiveness of the proposed model for the whole temperature range.

### 5.2. ASIC Results

When using the IPS measurement system, the distance is the final output, so we evaluate $U_{1}$ and the distance $L_{d}$ in the field experiments. In this subsection, the target is moved within the distance range of [3 mm, 7 mm] with a step of 0.04 mm for each temperature point. All the voltage values in this subsection are measured online from the evaluation PCB board (Figure 9b).

#### 5.2.1. ASIC Results of $U_{1}$

First, the intermediate variable $U_{1}$ is analyzed in the ASIC results. Based on the measured voltage $U_{1M}$, ${\hat{U}}_{1\mathrm{ML}}$ and ${\hat{U}}_{1\mathrm{MP}}$ denote the voltage computed by the LUT method and the proposed method, respectively. The difference between the measured $U_{1M}$ and the computed ${\hat{U}}_{1\mathrm{ML}}$ of the LUT method [28] is defined as $\Delta {\hat{U}}_{1\mathrm{ML}}$. The difference between the measured $U_{1M}$ and the computed ${\hat{U}}_{1\mathrm{MP}}$ of the proposed method is defined as $\Delta {\hat{U}}_{1\mathrm{MP}}$, shown as:

(10) $$\{\begin{array}{c} \Delta {\hat{U}}_{1\mathrm{ML}}\left(i,j\right)=U_{1M}\left(i,j\right)-{\hat{U}}_{1\mathrm{ML}}\left(i,j\right)\\ \Delta {\hat{U}}_{1\mathrm{MP}}\left(i,j\right)=U_{1M}\left(i,j\right)-{\hat{U}}_{1\mathrm{MP}}\left(i,j\right) \end{array}.$$

where the subscript i represents the sequence of the target distance from 3 mm to 7 mm with a step of 0.04 mm and the subscript j represents the temperature from $-55{\;}^{{}^{\circ}}C$ to $125{\;}^{{}^{\circ}}C$ with a step of $1{\;}^{{}^{\circ}}C$.

**Figure 12:**
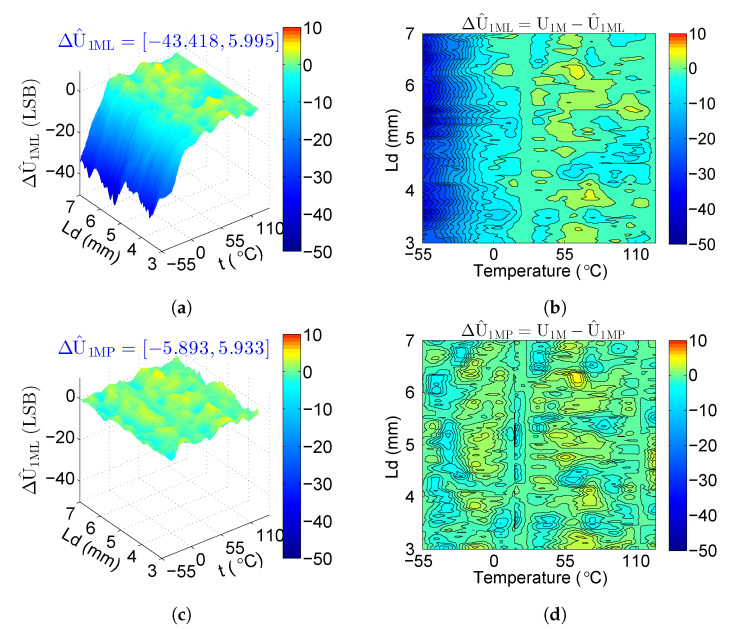
$\Delta {\hat{U}}_{1\mathrm{ML}}$ with different $\left\{L_{x},\;t\right\}$ displayed by: (**a**) 3D map; (**b**) contour map; $\Delta {\hat{U}}_{1\mathrm{MP}}$ with different $\left\{L_{x},\;t\right\}$ displayed by: (**c**) 3D map; (**d**) contour map (the blue color of the areas at a low temperature in (**a**,**b**) changes into green color in (**c**,**d**), which indicates that $\Delta {\hat{U}}_{1\mathrm{MP}}$ is closer to zero than $\Delta {\hat{U}}_{1\mathrm{ML}}$).

**Figure 13:**
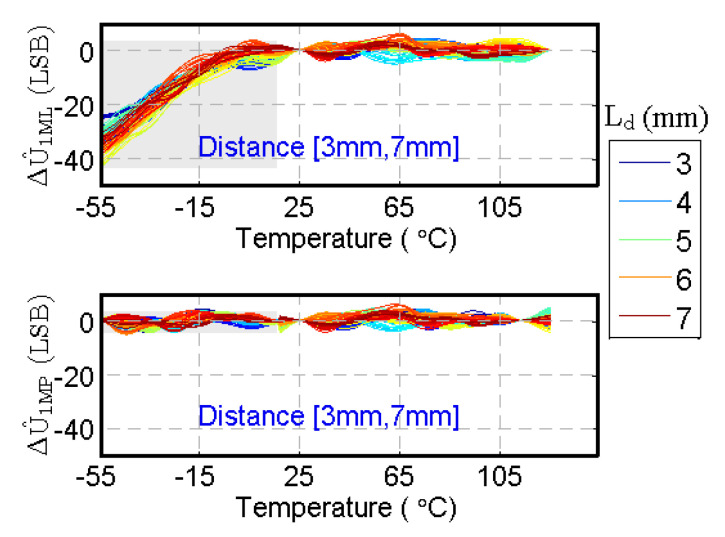
$\Delta {\hat{U}}_{1\mathrm{ML}}$ and $\Delta {\hat{U}}_{1\mathrm{MP}}$ with different $\left\{L_{x},t\right\}$ (at a low temperature, $\Delta {\hat{U}}_{1\mathrm{MP}}$ of the proposed method is closer to zero than $\Delta {\hat{U}}_{1\mathrm{ML}}$, which demonstrates that the proposed system can obtain more accurate calculation of $U_{1}$).

The proposed method generates more accurate estimated values, as clearly seen at a low temperature in Figure 12. The range of $\Delta {\hat{U}}_{1\mathrm{ML}}$ is [−43.418, 5.995] LSB and $\Delta {\hat{U}}_{1\mathrm{MP}}$ is [−5.893, 5.933] LSB, so $\Delta {\hat{U}}_{1\mathrm{MP}}$ is much less than $\Delta {\hat{U}}_{1\mathrm{ML}}$. The values of the colormap from top (−50) to bottom (10) are mapped to colors from dark blue through green to red, which is centered at zero to be colored green. The blue color of the areas at a low temperature in Figure 12a,b changes to green color in Figure 12c,d, which indicates that $\Delta {\hat{U}}_{1\mathrm{MP}}$ is closer to zero than $\Delta {\hat{U}}_{1\mathrm{ML}}$. We also show the covered curves of $\Delta {\hat{U}}_{1\mathrm{ML}}$ and $\Delta {\hat{U}}_{1\mathrm{MP}}$ for each distance in Figure 13. At a low temperature, $\Delta {\hat{U}}_{1\mathrm{MP}}$ of the proposed method is closer to zero than $\Delta {\hat{U}}_{1\mathrm{ML}}$, which demonstrates that the proposed system can obtain more accurate calculation of $U_{1}$.

Table 5 presents the detailed data analysis of $\Delta {\hat{U}}_{1\mathrm{ML}}$ and $\Delta {\hat{U}}_{1\mathrm{MP}}$. The values of $\Delta {\hat{U}}_{1\mathrm{MP}}$ are much less than $\Delta {\hat{U}}_{1\mathrm{ML}}$, especially the minimum values, thereby demonstrating that the proposed system can obtain more accurate distance estimation for all the cases, especially at a low temperature.

#### 5.2.2. ASIC Results of $D_{x}$

The estimated distance $D_{x}$ is the output of the IPS measurement system. Moving the target distance from 3 mm to 7 mm with a step of 0.04 mm, the estimated distance by the LUT method and the proposed method is computed, respectively.

The difference between the target distance $D_{x}$ and ${\hat{D}}_{\mathrm{xL}}$ of the LUT method [28] is defined as $\Delta {\hat{D}}_{\mathrm{xL}}$. The difference between $D_{x}$ and ${\hat{D}}_{\mathrm{xP}}$ of the method proposed in this paper is defined as $\Delta {\hat{D}}_{\mathrm{xP}}$, which can be shown as:

(11) $$\{\begin{array}{c} \Delta {\hat{D}}_{\mathrm{xL}}\left(i,j\right)=D_{x}\left(i,j\right)-{\hat{D}}_{\mathrm{xL}}\left(i,j\right)\\ \Delta {\hat{D}}_{\mathrm{xP}}\left(i,j\right)=D_{x}\left(i,j\right)-{\hat{D}}_{\mathrm{xP}}\left(i,j\right) \end{array}.$$

where the subscript i represents the sequence of the target distance from 3 mm to 7 mm with a step of 0.04 mm and the subscript j represents the temperature from $-55{\;}^{{}^{\circ}}C$ to $125{\;}^{{}^{\circ}}C$ with a step of $1{\;}^{{}^{\circ}}C$.

**Figure 14:**
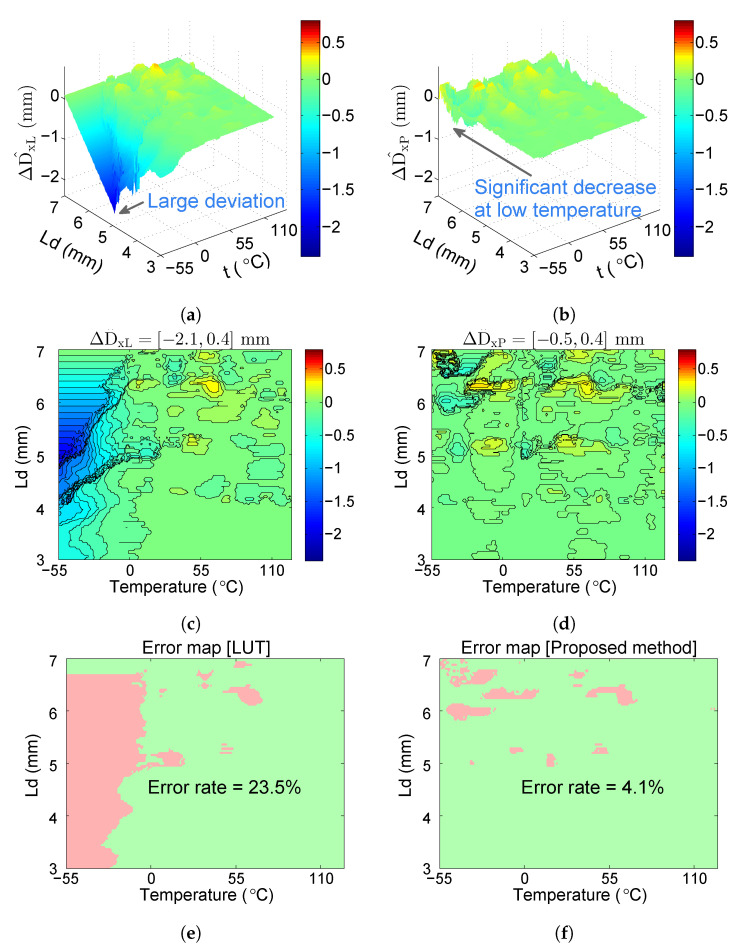
(**a**) ${\hat{D}}_{\mathrm{xL}}$ displayed by 3D map (the value of ${\hat{D}}_{\mathrm{xL}}$ is about −2.1 mm at a low temperature, which indicates the large distance deviation of the LUT method); (**b**) ${\hat{D}}_{\mathrm{xP}}$ displayed by 3D map (the value of ${\hat{D}}_{\mathrm{xP}}$ is close to zero mm at a low temperature, which shows that the proposed system can decrease the deviation of the distance estimation); (**c**) ${\hat{D}}_{\mathrm{xL}}$ displayed by contour map (${\hat{D}}_{\mathrm{xL}}$ is in the range of [−2.1, 0.4] mm, the large negative value of which results in the blue color of the areas at a low temperature); (**d**) ${\hat{D}}_{\mathrm{xP}}$ displayed by contour map (the blue color of the areas at a low temperature in (**c**) changes into green color in (**d**), which indicates that $\Delta {\hat{D}}_{\mathrm{xP}}$ is closer to zero than $\Delta {\hat{D}}_{\mathrm{xL}}$); (**e**) error map of ${\hat{D}}_{\mathrm{xL}}$ (the error rate of the LUT method is 23.5%); (**f**) error map of ${\hat{D}}_{\mathrm{xP}}$ (the error rate of our proposed method is 4.1%).

Figure 14 presents the differences of distance estimation ($\Delta {\hat{D}}_{\mathrm{xL}}$ and $\Delta {\hat{D}}_{\mathrm{xP}}$). The range of ${\hat{D}}_{\mathrm{xL}}$ is [−2.1, 0.4] mm, and the range of ${\hat{D}}_{\mathrm{xP}}$ is [−0.5, 0.4] mm, which shows that the proposed system can decrease the deviation of the distance estimation. Values of the colormap from top (−2.4) to bottom (0.8) are mapped to colors from dark blue through green to red, which is centered at zero to be colored green. The blue color of the areas at a low temperature in Figure 14a,b changes into green color in Figure 14d,e, which indicates that $\Delta {\hat{D}}_{\mathrm{xP}}$ is closer to zero than $\Delta {\hat{D}}_{\mathrm{xL}}$. Moreover, we set a 0.4 mm deviation tolerance for the correct distance estimation, which means the method works correctly when ${\hat{D}}_{\mathrm{xP}}\subseteq [D_{x}-0.2,D_{x}+0.2]\;\mathrm{mm}$. In Figure 14c,f, the green areas indicate the correct distance estimation, and the red areas indicate the incorrect distance estimation. The error rate is reduced from 23.5% (Figure 14c) to 4.1% Figure 14f. It can thus be concluded that the proposed system can improve the accuracy of distance estimation for the whole temperature range.

**Figure 15:**
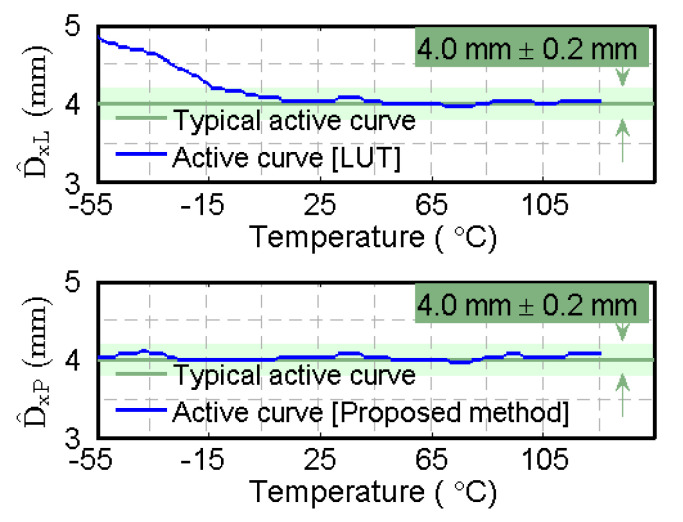
${\hat{D}}_{\mathrm{xL}}$ and ${\hat{D}}_{\mathrm{xP}}$ of 4 mm (the red curve of ${\hat{D}}_{\mathrm{xL}}$ provides an incorrect distance estimation at a low temperature; by contrast, the blue curve of ${\hat{D}}_{\mathrm{xP}}$ falls within the range of 4.0 mm±0.2 mm).

In our ASIC application, the most commonly used sensing distance is 4 mm. Figure 15 plots the estimated distance for 4 mm at different temperatures. At $-55{\;}^{{}^{\circ}}C$, when the target distance is 4 mm, the LUT method provides an incorrect distance estimation of 4.84 mm due to the interference of the low temperature. By contrast, the proposed method eliminates the effects of temperature, the estimated distances of which fall within the range of  4.0 mm±0.2 mm.

In Table 6, all estimated distances are within the range of [3.8, 4.2] mm with an average estimation of 4.034 mm, which demonstrates that a more accurate sensing distance has been obtained by the proposed system.

We present the performance summary and comparison with some publicly released references, which have the same application background in the field of aviation, shown in Table 7. The linear computation methods are used and only the simulation results are shown for [28,29]. The ASIC chips are implemented for [30] and our proposed method. The typical distance deviation of chips is decreased from 0.4 mm [30] to 0.12 mm, which demonstrates that the proposed system can obtain a more accurate sensing distance.

## 6. Conclusions

IPS suffers from temperature changes, which significantly decrease the measurement accuracy at low temperatures. Many proposed methods such as [27,28] achieve high accuracy at room temperature, but show large deviation at low temperatures. To improve the robustness for the temperature changing, we adopt the non-linear temperature compensation model, rather than the simple linear LUT method, to provide more accurate distance estimation within a wide temperature range compared to the previous work.

The innovations for high-accuracy measurement always increase the complexity of the ASIC implementation in the industrial fields, due to the limitation of hardware resources. We propose a serial dual-stage scheme to reduce the amount of online calculation and enable easy ASIC implementation, in which the offline stage performs a large amount of the computation and the online stage predicts the target distance with little hardware resource. Since the large amount of the parameter computation can be performed in the offline stage, the proposed system can guarantee both high accuracy and easy ASIC implementation.

This model is fabricated in a 0.18 μm CMOS process and packaged in a CQFN40. For the most commonly used sensing distance of 4 mm, the computed distance deviation of our ASIC chips falls within the range of [−0.2,0.2] mm in the temperature range from −55 °C to 125 °C, which demonstrates that the presented model could successfully achieve real-time and high-accuracy computation within a wide temperature range with low hardware resource consumption.

## Figures and Tables

**Table 1:** Temperature Coefficients (TCs).

TC	Value (ppm/${\;}^{{}^{\circ}}C$)	Description
$K_{R}$	0.000001	TC of R
$K_{R_{x}}$	0.00385	TC of $R_{x}$
$K_{\mathrm{RON}}$	0.0059	TC of $R_{\mathrm{ON}}$

**Table 2:** Typical resistance values.

Values	Type	Value at $25{\;}^{{}^{\circ}}C$	Description
$R_{0}$	Precision low TC/thick film metal	250	Rated value of the current limitation resistance R
$R_{x0}$	Copper coil (5 mH)	22	Rated value of the resistance of the inductor $R_{x}$
$R_{\mathrm{ON}0}$	N Type 2N7002	3.7	Rated value of the MOSFET turn on resistance $R_{\mathrm{ON}}$

**Table 3:** Feature parameters at various $L_{d}$.

i	$L_{d}$ (mm)	Feature Parameter
1	3	$P_{1}\left(1\right),P_{1}\left(2\right),P_{1}\left(3\right),P_{1}\left(4\right),P_{1}\left(5\right)$
2	3.004	$P_{2}\left(1\right),P_{2}\left(2\right),P_{2}\left(3\right),P_{2}\left(4\right),P_{2}\left(5\right)$
3	3.008	$P_{3}\left(1\right),P_{3}\left(2\right),P_{3}\left(3\right),P_{3}\left(4\right),P_{3}\left(5\right)$
…	…	…
101	7	$P_{101}\left(1\right),P_{101}\left(2\right),P_{101}\left(3\right),P_{101}\left(4\right),P_{101}\left(5\right),P_{101}\left(6\right)$

**Table 4:** Comparison of statistical values $\Delta {\hat{U}}_{1L}$ and $\Delta {\hat{U}}_{1P}$ (the computed ${\hat{U}}_{1P}$ by our method are closer to the theoretical $U_{1}$ than ${\hat{U}}_{1L}$ by the LUT method).

Voltage	Minimum	Maximum	Mean Value	Variance
$\Delta {\hat{U}}_{1L}$	−1.297 LSB	8.666 LSB	1.169 LSB	2.797 LSB
$\Delta {\hat{U}}_{1P}$	−0.015 LSB	0.106 LSB	0.011 LSB	0.020 LSB

**Table 5:** Comparison of statistical values $\Delta {\hat{U}}_{1\mathrm{ML}}$ and $\Delta {\hat{U}}_{1\mathrm{MP}}$ (the values of $\Delta {\hat{U}}_{1\mathrm{MP}}$ are much less than $\Delta {\hat{U}}_{1\mathrm{ML}}$, especially the minimum values, thereby demonstrating that the proposed system can obtain more accurate distance estimation).

Voltage	Minimum	Maximum	Mean Value	Variance
$\Delta {\hat{U}}_{1\mathrm{ML}}$	−43.418 LSB	5.995 LSB	−5.153 LSB	9.596 LSB
$\Delta {\hat{U}}_{1\mathrm{MP}}$	−5.893 LSB	5.933 LSB	0.206 LSB	1.509 LSB

**Table 6:** Comparison of the estimated distances when the target distance is 4 mm (${\hat{D}}_{P}$ are closer to 4 mm than ${\hat{D}}_{L}$, and all the values of ${\hat{D}}_{P}$ are within the range of [3.8, 4.2] mm, which demonstrates that a more accurate sensing distance was obtained by the proposed system).

Estimated Distance	Minimum	Maximum	Mean Value	Variance
${\hat{D}}_{L}$	3.960 mm	4.840 mm	4.165 mm	0.246 mm
${\hat{D}}_{P}$	3.960 mm	4.120 mm	4.034 mm	0.036 mm

**Table 7:** Performance summary and comparison with previous work.

Specifications	This Work	Sensors [29]	Sensors [28]	IEEE [30]	Honeywell [13]
Integrity of model	Full parasitic parameters	Partial parasitic parameters	N/A	Customized thermal resistor	N/A
Typical distance deviation	Chip test 0.12 mm	Simulation result −1.559 μm @5 mm, −55 °C	Simulation result −28 μm @5 mm, −55 °C	Chip test 0.4 mm (−55 °C vs. 70 °C)	N/A
Verification platform	Product results	Theoretical results (Table 3 and Table 4)	N/A	Product results	N/A
Computation method	Non-linear	Linear	Linear	N/A	N/A
Implementation technique	AD mixed ASIC	General purpose circuit module	General purpose circuit module	Analog ASIC	N/A
Current@28V	7.1 mA	6 mA	6 mA	4 mA	10 mA

## Abbreviations

The following abbreviations are used in this manuscript:

IPS	Inductive Proximity Sensor
LUT	Look-Up Table
CMOS	Complementary Metal Oxide Semiconductor
ASIC	Application-Specific Integrated Circuit
TC	Temperature Coefficient
PCB	Printed Circuit Board
LSB	Least Significant Bit
ADC	Analog-to-Digital Converter
AD	Analog Digital
EEPROM	Electrically Erasable Programmable Read only Memory
LDO	Low Dropout Regulator
POR	Power On Reset
